# Phosphorylation of hTERT at threonine 249 is a novel tumor biomarker of aggressive cancer with poor prognosis in multiple organs

**DOI:** 10.1002/path.5876

**Published:** 2022-03-23

**Authors:** Yoko Matsuda, Taro Yamashita, Juanjuan Ye, Mami Yasukawa, Keiko Yamakawa, Yuri Mukai, Mitsuhiro Machitani, Yataro Daigo, Yohei Miyagi, Tomoyuki Yokose, Takashi Oshima, Hiroyuki Ito, Soichiro Morinaga, Takeshi Kishida, Toshinari Minamoto, Shinji Yamada, Junko Takei, Mika K Kaneko, Motohiro Kojima, Shuichi Kaneko, Tsutomu Masaki, Masahiro Hirata, Reiji Haba, Keiichi Kontani, Nobuhiro Kanaji, Nobuyuki Miyatake, Keiichi Okano, Yukinari Kato, Kenkichi Masutomi

**Affiliations:** ^1^ Oncology Pathology, Department of Pathology and Host‐Defense, Faculty of Medicine Kagawa University Kita‐gun Japan; ^2^ Department of Gastroenterology Kanazawa University Graduate School of Medical Sciences Kanazawa Japan; ^3^ Division of Cancer Stem Cell National Cancer Center Research Institute Tokyo Japan; ^4^ Department of Medical Oncology and Cancer Center Shiga University of Medical Science Otsu Japan; ^5^ Center for Advanced Medicine against Cancer, Shiga University of Medical Science Otsu Japan; ^6^ Center for Antibody and Vaccine Therapy, Research Hospital, Institute of Medical Science Hospital, The University of Tokyo Tokyo Japan; ^7^ Kanagawa Cancer Center Research Institute Yokohama Japan; ^8^ Department of Pathology Kanagawa Cancer Center Yokohama Japan; ^9^ Department of Gastrointestinal Surgery Kanagawa Cancer Center Yokohama Japan; ^10^ Department of Thoracic Surgery Kanagawa Cancer Center Yokohama Japan; ^11^ Department of Hepato‐Biliary and Pancreatic Surgery Kanagawa Cancer Center Yokohama Japan; ^12^ Department of Urology Kanagawa Cancer Center Yokohama Japan; ^13^ Division of Translational and Clinical Oncology Cancer Research Institute, Kanazawa University Kanazawa Japan; ^14^ Department of Antibody Drug Development Tohoku University Graduate School of Medicine Sendai Japan; ^15^ Division of Pathology Exploratory Oncology Research and Clinical Trial Center, National Cancer Center Chiba Japan; ^16^ Department of Gastroenterology and Neurology Faculty of Medicine, Kagawa University Kita‐gun Japan; ^17^ Diagnostic Pathology Faculty of Medicine, Kagawa University Kita‐gun Japan; ^18^ Department of Thoracic, Breast and Endocrine Surgery Faculty of Medicine, Kagawa University Kita‐gun Japan; ^19^ Department of Internal Medicine, Division of Hematology, Rheumatology and Respiratory Medicine Faculty of Medicine, Kagawa University Kita‐gun Japan; ^20^ Department of Hygiene Faculty of Medicine, Kagawa University Kita‐gun Japan; ^21^ Department of Gastroenterological Surgery Faculty of Medicine, Kagawa University Kita‐gun Japan; ^22^ Department of Molecular Pharmacology Tohoku University Graduate School of Medicine Sendai Japan

**Keywords:** hTERT, phosphorylation, RdRP, pathology, poor prognosis

## Abstract

Recent evidence indicates that RNA‐dependent RNA polymerase (RdRP) activity of human telomerase reverse transcriptase (hTERT) regulates expression of target genes and is directly involved in tumor formation in a telomere‐independent manner. Non‐canonical function of hTERT has been considered as a therapeutic target for cancer therapy. We have previously shown that hTERT phosphorylation at threonine 249 (p‐hTERT), which promotes RdRP activity, is an indicator of an aggressive phenotype and poor prognosis in liver and pancreatic cancers, using two cohorts with small sample sizes with polyclonal p‐hTERT antibody. To clarify the clinical relevance of p‐hTERT, we developed a specific monoclonal antibody and determined the diagnostic and prognostic value of p‐hTERT in cancer specimens using a large cohort. A monoclonal antibody for phosphorylated hTERT (p‐hTERT) at threonine 249 was developed and validated. The antibody was used for the immunohistochemical staining of formalin‐fixed, paraffin‐embedded specimens from 1523 cases of lung, colon, stomach, pancreatic, liver, breast, and kidney cancers. We detected elevated p‐hTERT expression levels in cases with a high mitotic activity, high pathological grade, and high nuclear pleomorphism. Elevated p‐hTERT expression was an independent prognostic factor for lung, pancreatic, and liver cancers. Furthermore, p‐hTERT expression was associated with immature and aggressive features, such as adenosquamous carcinoma (lung and pancreas), invasive type of cancer (lung), high serum alpha‐fetoprotein level (liver), and triple‐negative status (breast). In conclusion, RdRP activity indicated by p‐hTERT expression predicts aggressive cancer phenotypes in various types of cancer. Thus, p‐hTERT is a novel biomarker for the diagnosis of aggressive cancers with a poor prognosis. © 2022 The Authors. *The Journal of Pathology* published by John Wiley & Sons Ltd on behalf of The Pathological Society of Great Britain and Ireland.

## Introduction

Cancer causes about one in six deaths worldwide and its incidence is increasing [1]. The incidence, morbidity, and mortality of cancer vary substantially according to the sex of patients and the organ of origin [1]. Cancer is also heterogeneous in terms of biological behavior, morphology, clinical responses to treatment, and prognosis, even for the same organ. Traditionally, this heterogeneity is considered to reflect variation in accumulated somatic mutations that promote malignant transformation [2]. However, several recent lines of experimental evidence indicate that the aged and cancer‐prone phenotype might represent the combined pathogenetic effects of the mutation load, epigenetic regulation, telomere dysfunction [3], altered stromal milieu [4], weakened stromal reaction, and decreased immune response against cancer cells [5, 6].

Telomeres are synthesized by telomerase, an enzyme composed of the catalytic protein subunit human telomerase reverse transcriptase (hTERT) and an RNA component. hTERT activity is readily detected in normal embryonic/pluripotent stem cells and in the majority of human cancer cells, suggesting that it confers cell immortality. However, the correlation between the enzymatic activity of hTERT and *hTERT* (*TERT*) expression levels in cancer remains elusive [7]. Furthermore, there is strong evidence for the importance of *hTERT* expression in cancer stem cells [8]. The cancer stem cell hypothesis predicts that a subset of tumor cells possesses stem cell features in terms of self‐renewal and differentiation capacity. Cancer stem cells are highly tumorigenic, metastatic, resistant to treatment, and correlated with poor prognosis in various solid tumors [9], and these features cannot be explained by the canonical function of hTERT in the maintenance of telomeres. Specific antibodies against hTERT have been developed to evaluate hTERT protein levels in clinical specimens [10, 11]. However, no obvious correlation between hTERT expression levels and prognosis has been observed in immunohistochemical analyses of clinical samples [12, 13].

We recently found that hTERT demonstrates RNA‐dependent RNA polymerase (RdRP) activity [14]. RdRP is an enzyme that catalyzes the replication of RNA from an RNA template and is an essential protein of RNA viruses. hTERT‐RdRP activity generated dsDNAs that are processed to siRNAs for the purpose of downregulating gene expression [14], and affected gene expression by affecting RNA levels [15]. hTERT‐RdRP is involved in tumor formation in a telomere‐independent manner [16]. The non‐canonical function of hTERT has been considered as an effective target for cancer therapy [17]. hTERT forms a complex with Brahma‐related gene 1 (BRG1) and nucleostemin (NS) [15] and maintains cancer stem cell properties via RdRP activity [18]. Furthermore, we recently demonstrated that hTERT is phosphorylated at threonine 249 (Thr249) by the serine/threonine kinase CDK1, and this phosphorylation event works as a molecular switch for the RdRP activity of hTERT without affecting telomerase activity. hTERT‐RdRP activity was involved in the expression of various genes such as Forkhead box O4 (*FOXO4*), a tumor suppressor gene, by preventing proper cell cycle. Most importantly, the abrogation of hTERT phosphorylation and RdRP activity significantly inhibits tumorigenesis *in vivo* by regulating the expression of *FOXO4*. hTERT phosphorylation at Thr249 is correlated with poor survival outcomes in pancreatic and liver cancers [16]. Moreover, eribulin mesylate, a specific inhibitor of hTERT‐RdRP activity, inhibited ovarian cancer cell growth *in vitro* [19] and glioblastoma cell growth in the subcutaneous and intracranial xenograft mouse [20]. The expression level of hTERT was correlated to sensitivity for eribulin mesylate [19]. While eribulin was originally identified as an inhibitor of microtubular synthesis that is essential for cell division, and therefore inhibition of hTERT‐RdRP may affect the microtubular network, these data reveal the value of hTERT‐RdRP for the diagnosis of cancer with a poor prognosis and as a useful marker to predict response to treatment.

Here, we successfully developed a mouse monoclonal antibody that specifically recognizes hTERT phosphorylated at Thr249. This antibody provides the first effective tool for the visualization of hTERT‐RdRP in cancer using formalin‐fixed, paraffin‐embedded tissues. Using this antibody, we analyzed 1523 cancer specimens (including lung, colorectal, stomach, pancreatic, liver, breast, and kidney cancer specimens) to identify the clinicopathological characteristics of hTERT‐RdRP‐active cancer.

## Materials and methods

### Generation of hybridoma‐producing phospho‐specific monoclonal antibody, TpMab‐3

Details are provided in Supplementary materials and methods. To generate a monoclonal antibody against phosphorylated threonine 249 of hTERT (TpMab‐3), hTERT phosphopeptide 244CEPERpTPVGQG254 was used to immunize mice. Hybridoma was generated as previously reported [16]. Culture supernatants were screened by enzyme‐linked immunosorbent assay (ELISA) for the detection of the p‐hTERT peptide and wild‐type TERT peptide as previously reported [16]. Clone TpMab‐3 (IgG_1_, kappa), which is specific for the p‐hTERT peptide, was finally established.

### Cell culture and mitotic cell synchronization

The human cell lines used in the present study are listed in supplementary material, Table S1. Cells were induced to enter the mitotic phase for the enrichment of phospho‐hTERT and RdRP activity following a previously described method [16, 21]. In brief, cells were cultured with standard medium, switched to medium containing 2.5 mm thymidine (Nacalai Tesque, Inc, Kyoto, Japan), and incubated for 24 h. Six hours after release, cells were incubated in medium containing 0.1 μg/ml nocodazole (Sigma‐Aldrich, St Louis, MO, USA) for 16 h. Mitotic cells were retrieved by mitotic shake‐off. Cells arrested in mitosis with nocodazole were confirmed by immunoblotting using anti‐phospho‐histone H3 (Ser10) antibodies.

### siRNA transfection

HeLa cells were transfected with siRNAs using Lipofectamine 2000 (Thermo Fisher Scientific Inc, Waltham, MA, USA) as previously reported [16]. After 48 h of incubation, cells were treated with 0.1 μg/ml nocodazole for 16 h. The sequences of siRNAs against hTERT were as follows: TERT siRNA#1, GUGUCUGUGCCCGGGAGAATT; #2, GCAUUGGAAUCAGACAGCATT. MISSION siRNA Universal Negative Control #1 (Sigma‐Aldrich) was used as a negative control.

### Detection of phosphorylated hTERT by immunoprecipitation

Details are provided in Supplementary materials and methods. Anti‐hTERT mouse monoclonal antibodies (clones 10E9‐2 and 2E4‐2) were generated, and the specificity was evaluated as reported previously [15]. Anti‐hTERT mouse mAb (clone 2E4‐2) and Mouse TrueBlot ULTRA: Anti‐Mouse Ig HRP (Rockland, Gilbertsville, PA, USA) were used for immunoblotting to detect whole‐hTERT proteins [21]. Anti‐phospho‐hTERT mouse monoclonal Ab (clone TpMab‐3) and Mouse TrueBlot ULTRA: Anti‐Mouse Ig HRP (Rockland) were used to detect phosphorylated hTERT. For λ phosphatase treatment, the bead suspension with immune complexes was treated with 2000 U of λ protein phosphatase (λ‐PPase) (Bio Academia, Osaka, Japan) and 2 mm MnCl_2_ in λ‐PPase reaction buffer [50 mm Tris–HCl (pH 7.6), 100 mm NaCl, 2 mm DTT, 100 μm EDTA, and 0.01% Brij 35] and incubated at 30 °C for 30 min.

### Immunoprecipitation–RdRP assay

Details are provided in Supplementary materials and methods. hTERT was immunoprecipitated from human cell lines as described previously with an anti‐hTERT mAb (clone 10E9‐2) [15, 16, 21]. The sequence of the RNA template was as follows: 5’‐GGGAUCAUGUGGGUCCUAUUACAUUUUAAACCCA‐3’.

### Quantitative PCR

Total RNA was isolated from human cell lines using the RNeasy Mini Kit (Qiagen, Hilden, Germany) and treated with the RNase‐Free DNase Set (Qiagen). cDNAs were synthesized from total RNA using SuperScript IV VILO Master Mix (Thermo Fisher Scientific Inc), amplified by PCR using the TaqMan Gene Expression Assay (TERT, Hs00972650_ml; 18S rRNA, 4310893E, Thermo Fisher Scientific), and analyzed with the StepOnePlus Real‐Time PCR System (Thermo Fisher Scientific).

### Patients

The study was conducted in accordance with the principles embodied in the Declaration of Helsinki, 2013. All experiments were approved by the ethics committees of Kagawa University (permit number 2019‐209), the Human Genome/Gene Analysis Ethics Committee of Kanazawa University (approval No. 181), and Kanagawa Cancer Center (approval No. 177). Surgically resected tissues were obtained from consecutive case series that underwent surgical treatment at Kagawa University Hospital (pancreas, between 2008 and 2020; breast, 2005–2011) and Kanazawa University Hospital (stomach, 2001–2009; liver, between 2005 and 2014). Tissue microarray (TMA) specimens were obtained from the National Cancer Center Hospital East (liver, between 2010 and 2019), Kanazawa University Hospital (colon, between 1997 and 2005), and Platform of Supporting Cohort Study and Biospecimen Analysis (http://cohort.umin.jp/english/index.html) (lung, stomach, pancreas, kidney). All tumors were taken at diagnosis. We adhered to the REMARK criteria [22].

### Tissue processing

For preparing whole slides, formalin‐fixed and paraffin‐embedded (FFPE) tissues from the invasive and advanced lesion which showed representative pathological histological features of tumors were used. TMA specimens were made from the archives of FFPE tissues used for routine histopathologic diagnosis at the pathology department of Kanagawa Cancer Center. In brief, an experienced genitourinary pathologist centrally reviewed the hematoxylin and eosin (H&E)‐stained slides and marked the areas to be punched out for a TMA. Subsequently, FFPE tissue cores (2 mm in diameter) corresponding to the marked areas on the H&E slides were obtained using manual tissue microarrayers (KIN‐3 model; AZUMAYA Inc, Tokyo, Japan/Mini Core; ALPHELYS, Plaisir, France). For each case, two representative, independent tumor areas and a non‐neoplastic cortical area were processed. Pathology specimens were used for lung cancer (TMA, *n* = 342), colon cancer (TMA, *n* = 117), stomach cancer (whole slide, *n* = 80; TMA, *n* = 122), pancreatic cancer (whole slide, *n* = 53; TMA, *n* = 199), liver cancer (whole slide, *n* = 194; TMA, *n* = 199), breast cancer (whole slide, *n* = 85), and kidney cancer (TMA, *n* = 132) (supplementary material, Table S2). [16]. Whole slides of liver cancer (*n* = 194) were an expansion of the cohort published previously [16], and pancreatic cancer (*n* = 252) and TMA of liver cancer (*n* = 199) were an independent group of previous cohorts.

The tissues were sliced serially into sections (3 μm thick) for H&E and immunohistochemical staining and fluorescence *in situ* hybridization (FISH). Pathological specimens were diagnosed by our pathologists based on the World Health Organization Classification of Tumours [23, 24, 25, 26]. Immunohistochemical staining was performed for the detection of p40 (prediluted; Nichirei Bioscience Inc, Tokyo, Japan), p63 (7JUL, prediluted; Leica Biosystems, Wetzlar, Germany), cytokeratin 5/6 (D5/16B4, prediluted; Agilent Technologies, Inc, Santa Clara, CA, USA), napsin A (IP64, 1:100 dilution; Leica Biosystems), and TTF1 (8G7G3/1, 1:50 dilution; Agilent Technologies, Inc) to distinguish adenocarcinoma from squamous cell carcinoma for all lung cancer specimens. Immunohistochemical staining of estrogen receptor (6F11, prediluted; Leica Biosystems), progesterone receptor (16, prediluted; Leica Biosystems), and HER2 (CB11, prediluted; Leica Biosystems) was performed for all breast cancer cases to identify triple‐negative breast cancer. The pathological stage was diagnosed based on the *TNM Classification of Malignant Tumors*, 7th edition [27].

### Immunohistochemical staining for phosphorylated hTERT and Ki67

Tissue specimens were immunostained using the Ventana Discovery Staining System (Roche, Basel, Switzerland) and the DISCOVERY ChromoMap DAB Kit (Roche) according to the manufacturer's instructions. The tissue sections were preheated with CC1 (pH 9.0, Roche) for 30 min at 100 °C. They were then incubated with the mouse monoclonal anti‐p‐hTERT antibody (1:500 dilution) or Ki67 (MIB1, 1:100 dilution; Agilent Technologies, Inc) for 12 h at 25 °C. Incubation with OmniMap anti‐mouse HRP‐conjugated multimer secondary antibody (Roche) was performed for 32 min at 25 °C. We performed immunohistochemical staining of mouse IgG1 (isotype control; Abcam, Cambridge, UK) under the same conditions as p‐hTERT and Ki67 staining, and confirmed negative staining. The proportion of cancer cells with positively stained nuclei was analyzed at ×200 by the authors (YMa, JY, KY, or TY) [16]. The percentage of p‐hTERT and Ki67 expression was scored every 10% as follows: 0–9%, 0; 10–19%, 10; 20–29%, 20; and so on.

### Pathological assessment

The authors (YM, JY, or KY) reviewed H&E‐stained specimens for pathological assessments. Mitotic counts in 10 high‐power fields (HPF) were obtained using H&E‐stained specimens at a magnification of ×400. The mitotic count was scored based on the WHO classification of breast tumors (field diameter, 0.54 mm; score 1, ≤8 mm; score 2, 9–16 mm; score 3, ≥17 mm) [23]. Ki67 immunostaining was performed to evaluate proliferative activity using breast cancer specimens to confirm the relationship between the mitotic count and Ki67 index.

Pathological grade was based on the *TNM Classification of Malignant Tumors*, 7th edition (G1, well differentiated; G2, moderately differentiated; G3, poorly differentiated; G4, undifferentiated; GX, cannot be assessed) [27].

The nuclear score was assessed at 200× magnification based on the WHO classification of breast tumors (score 1, small, regular, uniform cells; score 2, moderate increase in size and variability; score 3, marked variation) [23].

### Quantitative fluorescence *in situ* hybridization for the analysis of telomere length

Slides were processed by FISH, as previously reported [28]. In brief, tissue sections were hybridized with 200 nm PNA probes for the telomere (telo C‐Cy3 probe, 5’‐CCCTAACCCTAACCCTAA‐3’; Panagene, Daejeon, Korea) and the centromere (Cenp1‐FITC probe, 5’‐CTTCGTTGGAAACGGGGT‐3’; Panagene) for 3 min at 80 °C and then for 1 h at room temperature. The nuclei were stained with DAPI (Molecular Probes, Eugene, OR, USA). FISH images were captured using a fluorescence microscope (FSX100; Olympus, Tokyo, Japan) at ×800 magnification.

ImageJ (version 1.53a, Wayne Rasband, National Institutes of Health, Bethesda, MD, USA; modified by the plug‐in AsKey, Kagawa, Japan) was used to estimate the red, green, and blue intensities of individual nuclei. As an entire nucleus will not necessarily be captured within any given tissue section, the total corrected telomere signal for each nucleus was normalized by the corresponding integrated optimal density of the centromere as the telomere/centromere ratio. Over 100 cells were analyzed for each sample. As a control for variation in sample preparation, FISH was also performed on sections of a block preparation of a cultured cell strain, HFL‐1 (with a population doubling level of 20). The normalized telomere signals for each case were calculated as follows: [median value of telomere/centromere ratio of target cells]/[median value of telomere/centromere ratio of control HFL‐1 cells]. Patients were divided into two groups based on [normalized telomere length of cancer cells]/[normalized telomere length of fibroblasts], with a cut‐off value of 1.2 to obtain cases with short telomeres (under 1.2) and cases with long telomere lengths (over 1.2).

### Statistical analysis

Two groups were compared by the unpaired *t*‐test, χ^2^ test, and Fisher's exact test. Three or more groups were compared by an analysis of variance (ANOVA) and Tukey's test. Correlations were assessed by Pearson's and Spearman correlation coefficients. Survival was analyzed by Kaplan–Meier curves and a Cox proportional hazard model. Univariate and multivariate analyses were performed using sex, age (cut‐off value: 65 years old), p‐hTERT expression (cut‐off value: median), and TNM stage. The level of significance was set at *p* < 0.05 for all analyses. Statistical analyses were performed using JMP Pro 14 (SAS Institute Inc, Cary, NC, USA) and Statistical Package for the Social Sciences version 22 (IBM Corp, New York, NY, USA).

## Results

### Characterization and validation of the mAb specific for phosphothreonine 249 of hTERT

**Figure 1:**
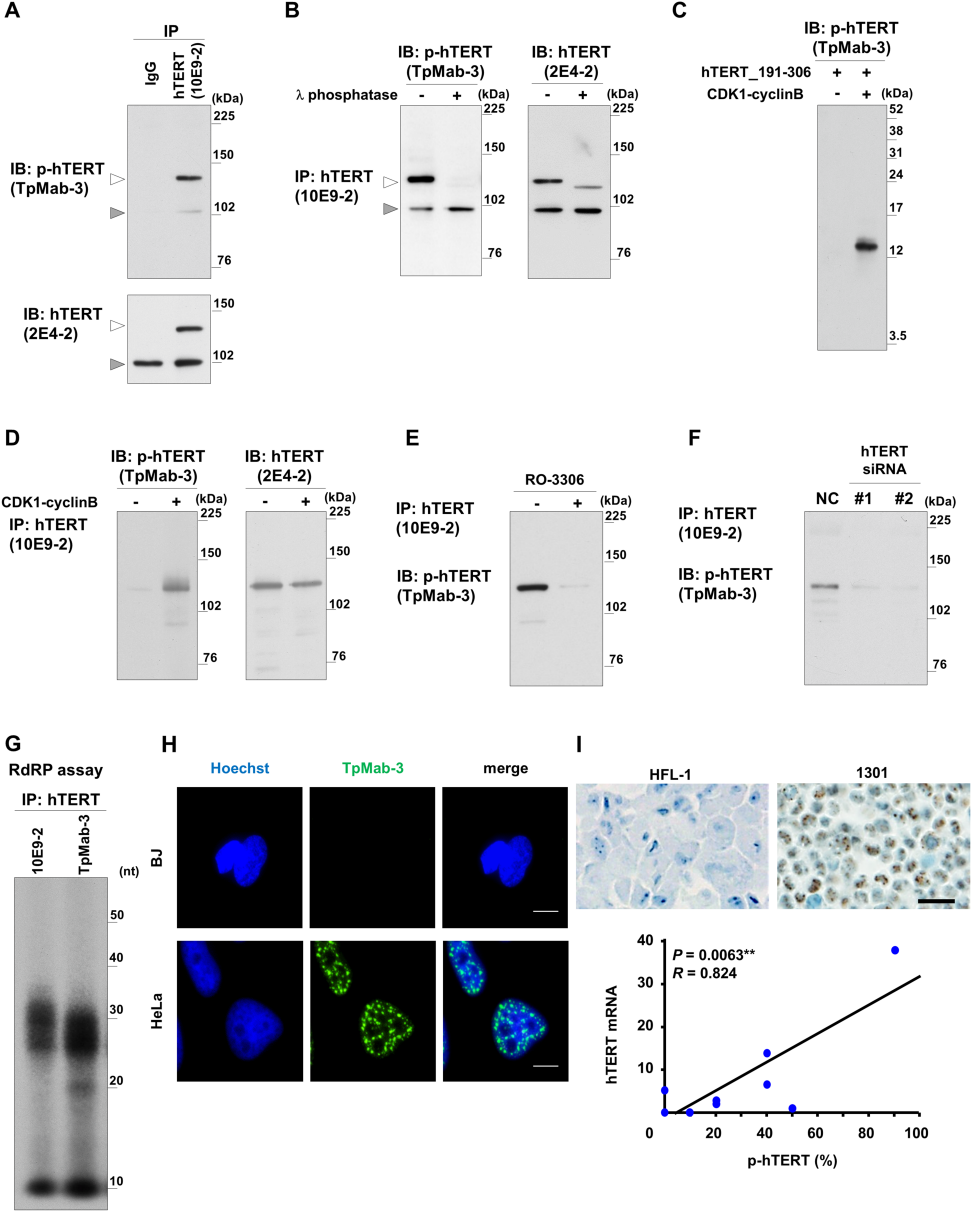
Validation of a monoclonal antibody (TpMab‐3) specific for hTERT phosphorylated at Thr249. (A) Detection of endogenous p‐hTERT from HeLa cells treated with nocodazole to synchronize cells in mitosis. Endogenous hTERT was immunoprecipitated by an anti‐hTERT mouse mAb (10E9‐2) and detected by an anti‐p‐hTERT mouse mAb (TpMab‐3) or an anti‐hTERT mouse mAb (2E4‐2). Mouse IgG was used as an isotype control for immunoprecipitation. (B) hTERT immunoprecipitated from the cells was treated with λ‐phosphatase and detected by an anti‐p‐hTERT mouse mAb (TpMab‐3) and an anti‐hTERT mouse mAb (2E4‐2). White arrowheads (in panels A and B) indicate hTERT signals and the band at 102 kDa (gray arrowhead) is a nonspecific signal from the secondary antibody [16]. (C) The recombinant hTERT fragment proteins (191–306 a.a.) [16] were phosphorylated by CDK1–cyclin B *in vitro* and phosphorylation of hTERT at threonine 249 by CDK1–cyclin B was confirmed by TpMab‐3. (D) Same as panel C, *in vitro* kinase assay was performed *in vitro* using the recombinant hTERT full‐length proteins [29]. Phosphorylation of hTERT at threonine 249 by CDK1–cyclin B was confirmed by TpMab‐3 and 2E4‐2. (E) Cells were treated with a CDK1 inhibitor (RO‐3306). hTERT was immunoprecipitated by 10E9‐2 and detected by an anti‐p‐hTERT mouse mAb (TpMab‐3, Thr249). (F) hTERT immunocomplex was immunoprecipitated (10E9‐2) from cells transfected with two different siRNAs specific for hTERT or siNC, followed by nocodazole treatment. The proteins were detected by TpMab‐3. (G) RdRP assay using hTERT immunoprecipitated with anti‐hTERT mouse mAb (clone 10E9‐2) or anti‐phospho‐hTERT (TpMab‐3). (H) Immunofluorescence staining of p‐hTERT in cell lines (BJ, human fibroblast; HeLa, human cancer cells). Bar: 10 μm. (I) Immunohistochemical staining of p‐hTERT in cell lines (HFL‐1, p‐hTERT‐negative human fibroblast; 1301, p‐hTERT‐positive human leukemia cells). Bar: 20 μm. Correlations between *TERT* mRNA and TpMab‐3 expression were determined using nine human cell lines. IB, immunoblotting; IP, immunoprecipitation.

To investigate the clinical significance of phosphothreonine 249 of hTERT, we generated a monoclonal antibody (TpMab‐3) directed against hTERT phosphorylated at Thr249. In combination with validated monoclonal antibodies specific for total hTERT proteins (clones 10E9‐2 and 2E4‐2) [15], we comprehensively validated the specificity of TpMab‐3 by a standard validation method [16]. In brief, we performed immunoprecipitation followed by immunoblotting (Figure 1A), treatment with λ‐phosphatase to confirm that the signals are phosphothreonine‐specific (Figure 1B), and an *in vitro* kinase assay reconstituted by CDK1–cyclin B proteins with two different versions of recombinant hTERT proteins [hTERT amino acids 191–306 [16] (Figure 1C) and full‐length hTERT protein [29] (Figure 1D)]. In addition, as a reciprocal experiment, we further validated the specificity of TpMab‐3 by treatment with the CDK‐1 inhibitor RO‐3306 (Figure 1E). The suppression of hTERT by siRNAs specific for *hTERT* [18, 29] further confirmed that the signals are hTERT‐specific (Figure 1F). More specifically, once we had suppressed hTERT by siRNAs specific for *hTERT* [18, 29] followed by IP–IB, we were unable to detect hTERT signal by TpMab‐3, indicating that the signals are hTERT‐specific. Taken together, these data indicate that the signals are specific for phosphothreonine 249 of hTERT protein. Given that the phosphorylation of Thr249 is necessary for hTERT‐mediated RdRP activity, we monitored whether TpMab‐3 is able to recover RdRP activity *in vitro* (Figure 1G). In each case, TpMab‐3 identified phosphothreonine 249 (p‐hTERT), confirming the sensitivity and specificity of the antibody. Thus, we concluded that TpMab‐3 is effective for immunohistochemical analyses. TpMab‐3 revealed expression in nuclei of HeLa cells but not in normal fibroblast BJ cells (Figure 1H). In addition, in several human cell lines, we found that the level of p‐hTERT was associated with the mRNA level of *hTERT* (*p* = 0.0063, *R* = 0.651; Figure 1I).

### Phosphorylation of hTERT at threonine 249 occurs in various cancers

**Figure 2:**
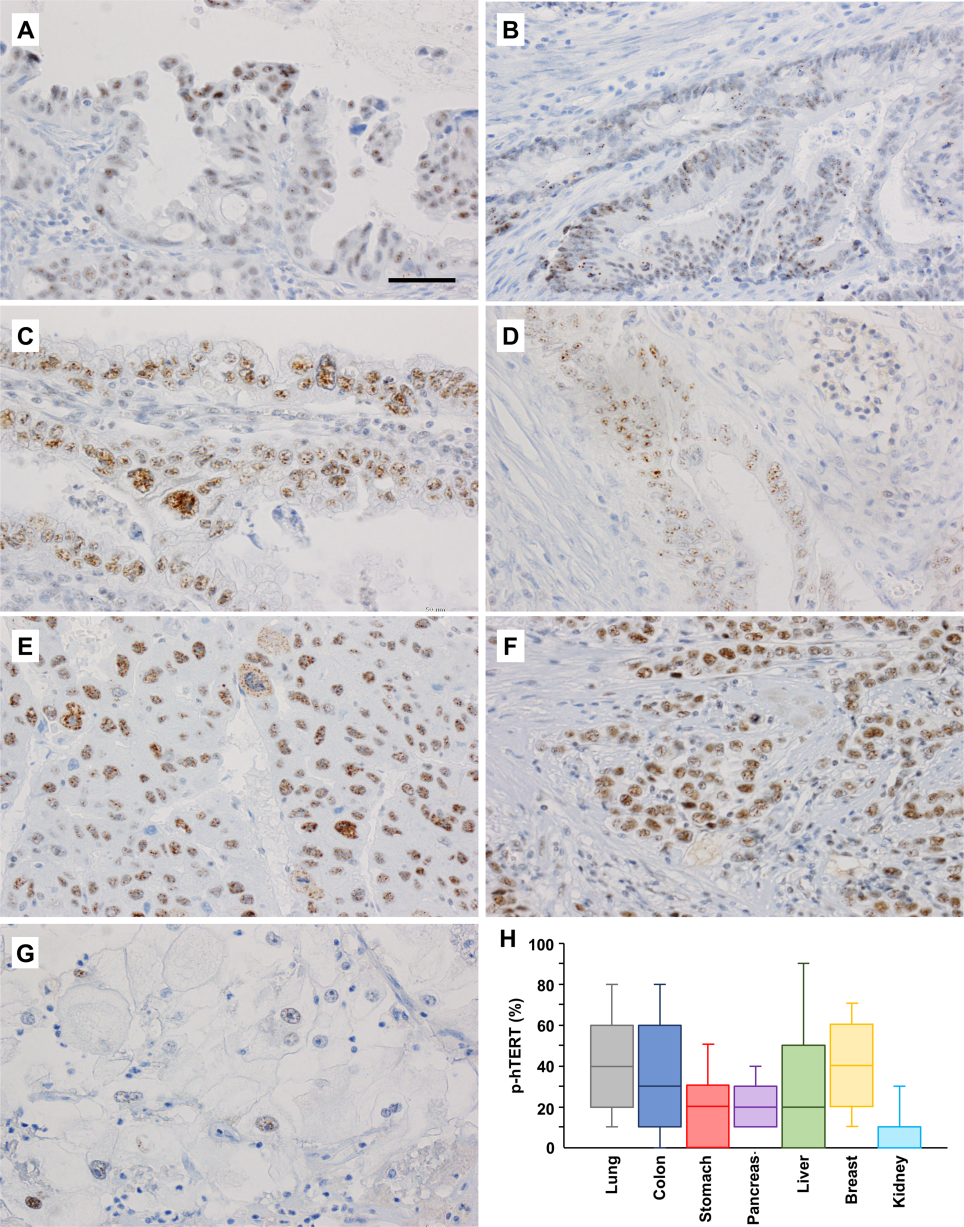
Variation in p‐hTERT expression among cancers. (A) Lung adenocarcinoma, (B) colon adenocarcinoma, (C) gastric adenocarcinoma, (D) pancreatic adenocarcinoma, (E) hepatocellular carcinoma, (F) breast adenocarcinoma, and (G) renal cell carcinoma. Original magnification: ×400. Bar: 50 μm. (H) Box plot of p‐hTERT expression in cancers in various organs.

The expression of p‐hTERT (Thr249) was detected in cancer cell nuclei (Figure 2A–G). The frequencies of p‐hTERT‐positive cancer cells varied depending on cancer type and organ (Figure 2H). No positive associations were found between the expression of p‐hTERT and TNM stage (supplementary material, Figure S1), age (supplementary material, Figure S2), or sex (supplementary material, Figure S3).

**Figure 3:**
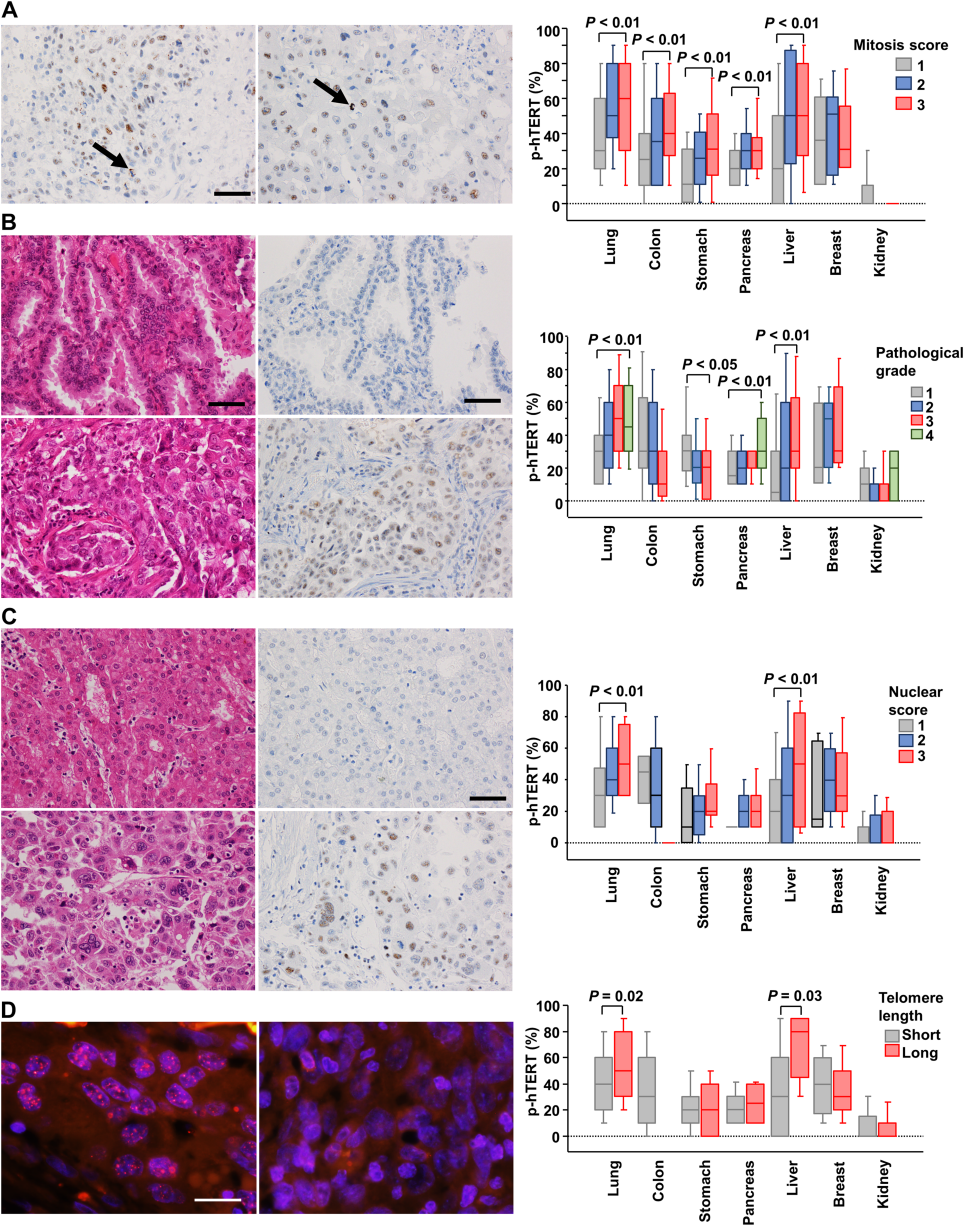
Associations of p‐hTERT expression with mitosis score, pathological grade, nuclear grade, and telomere length. (A) p‐hTERT expression in mitotic cells (arrows). The plot shows expression of p‐hTERT for each mitosis score in cancers from various organs. (B) Upper panels: lung adenocarcinoma case classified as pathological grade 1, well‐differentiated adenocarcinoma. Lower panels: lung adenocarcinoma case classified as pathological grade 3, poorly differentiated adenocarcinoma. The plot shows expression of p‐hTERT for each pathological grade in various organs. (C) Upper panels: hepatocellular carcinoma case with nuclear score 1; small, regular, uniform cells. Lower panels: hepatocellular carcinoma case with nuclear score 3, marked variation. The plot shows p‐hTERT for each nuclear score in various organs. (A–C) Original magnification: ×400. Bar: 50 μm. Spearman's rank correlation coefficient. (D) Left panel: lung adenocarcinoma case with a long telomere length. Right panel: lung adenocarcinoma case with a short telomere length. FISH images of telomeres (red), centromeres (green), and DAPI (blue); original magnification: ×800. Bar: 20 μm. Patients were divided into two groups based on [normalized telomere length of cancer cells]/[normalized telomere length of fibroblasts] with a cut‐off value of 1.2 to obtain cases with short telomeres (under 1.2) and cases with long telomere lengths (over 1.2). The plot shows expression of p‐hTERT for Short and Long telomere length groups in various organs. Student's *t*‐test.

Next, we analyzed the link between p‐hTERT expression and pathological characteristics typically associated with aggressive phenotypes in cancer, including mitosis score, pathological grade [30], and nuclear score [23, 27, 31] (Figure 3A–C and supplementary material, Table S2). Populations of p‐hTERT‐positive cancer cells exhibited a positive association with mitosis score in cancers of the lung (*p* < 0.0001), colon (*p* = 0.0068), stomach (*p* < 0.0001), pancreas (*p* = 0.0010), and liver (*p* < 0.0001) (Figure 3A). We confirmed that the number of mitosis per 10 HPF was positively correlated to the Ki67 index (*p* < 0.0001; *R* = 0.373; supplementary material, Figure S4).

p‐hTERT expression increased as the pathological grade increased in cancers of the lung (*p* < 0.0001), pancreas (*p* < 0.0001), and liver (*p* = 0.0005; Figure 3B). We observed elevated p‐hTERT expression in patients with a high nuclear score in cancers of the lung (*p* < 0.0001) and liver (*p* < 0.0001; Figure 3C). These results indicate that p‐hTERT was highly expressed in cancers with aggressive phenotypes, such as cancers with high proliferative activity, a high pathological grade, and severe nuclear atypia. The phosphorylation event at threonine 249 is essential for RdRP activity but does not affect telomerase activity, and we have reported that phosphorylation is not involved in telomere maintenance in several cancer cell lines [16]. To validate this result using clinical specimens, we evaluated the correlation between p‐hTERT expression and telomere length, as determined by FISH, in 1399 cancer specimens. In lung and liver cancers, p‐hTERT expression levels were higher in tissue sections with long telomeres than in sections with short telomeres (lung, *p* = 0.0172; liver, *p* = 0.0259; Figure 3D).

**Figure 4:**
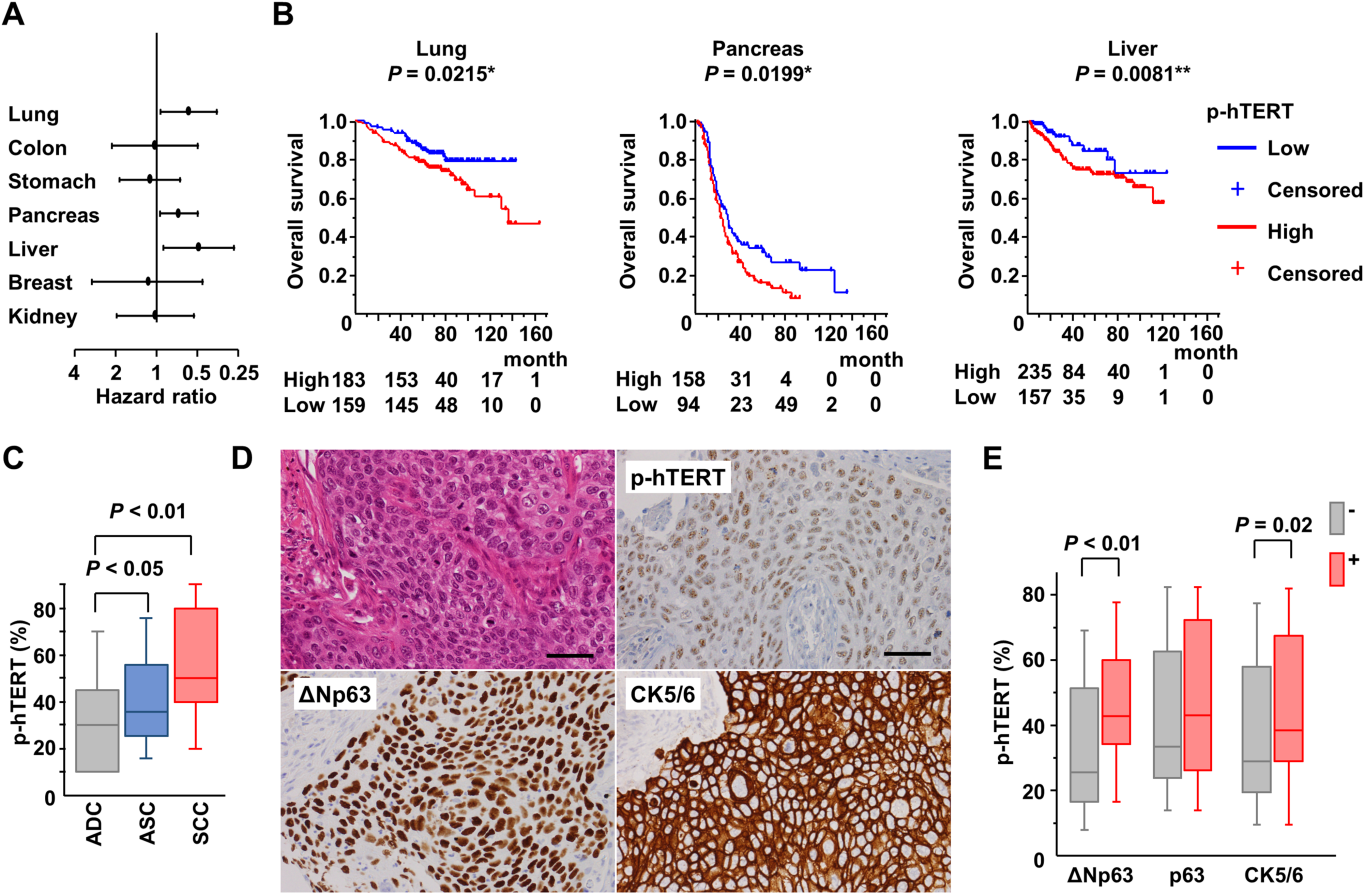
p‐hTERT expression is a potential biomarker for poor prognosis in lung, pancreatic, and liver cancers. (A) Hazard ratio for overall survival. (B) Kaplan–Meier analysis for overall survival. Cut‐off value for p‐hTERT expression was 40% for lung, 20% for pancreatic, and liver cancer according to the median expression. The number of patients at risk is shown below the graph. *p < 0.05 and **p < 0.01 by log‐rank test. (C) p‐hTERT expression of adenocarcinoma (ADC), adenosquamous carcinoma (ASC), and squamous cell carcinoma (SCC). (D) Lung squamous cell carcinoma case. Original magnification: ×400. Bar: 200 μm. (E) Expression levels of ΔNp63, p63, and cytokeratin 5/6 were associated with p‐TERT. Red box, positive for ΔNp63, p63, or cytokeratin 5/6; gray box, negative. Student's *t*‐test.

Univariate Cox regression analysis indicated that high p‐hTERT expression was associated with short overall survival in cancers of the lung [hazard ratio (HR) 0.58, 95% confidence interval (CI) 0.36–0.93, *p* = 0.024], pancreas (HR 0.69, 95% CI 0.5–0.94, *p* = 0.021), and liver (HR 0.49, 95% CI 0.27–0.85, *p* = 0.019; Figure 4A and Table 1). Multivariate Cox regression analysis indicated that high p‐hTERT expression and TNM stage are independent risk factors for short overall survival in lung, pancreatic, and liver cancers (Table 1), and disease‐free survival in pancreatic and liver cancers (Table 2). Patients with a high level of p‐hTERT showed an association with pathological type in lung cancer; high mitosis score in lung, colon, stomach, pancreas, and liver cancers; high pathological grade in lung, pancreas, and liver cancers; and high nuclear score in lung cancers (supplementary material, Table S2), suggesting that high p‐hTERT expression contributes to the proliferation and highly aggressive morphological features of those cancers.

**Table 1:** Univariate and multivariate analyses of overall survival

Univariate		Lung		Colon		Stomach		Pancreas		Liver		Breast		Kidney	
		HR	*P* value	HR	*P* value	HR	*P* value	HR	*P* value	HR	*P* value	HR	*P* value	HR	*P* value
Sex	Male	1		1		1		1		1		ND		1	
	Female	1.73 (1.07–2.79)	0.0243*	1.11 (0.55–2.21)	0.7767	0.97 (0.55–1.69)	0.9021	0.82 (0.61–1.10)	0.1887	0.99 (0.52–1.91)	0.9816			1.46 (0.69–3.08)	0.3201
Age (years)	≤65	1		1		1		1		1		1		1	
	>65	0.70 (0.44–1.12)	0.1407	0.60 (0.28–1.30)	0.1950	0.93 (0.56–1.55)	0.7894	1.02 (0.75–1.38)	0.9100	1.12 (0.68–1.84)	0.6663	0.87 (0.34–2.24)	0.7778	0.78 (0.41–1.49)	0.4541
hTERT	Low	1		1		1		1		1		1		1	
	High	0.58 (0.36–0.93)	0.0240*	1.03 (0.50–2.13)	0.9453	1.12 (0.67–1.87)	0.6548	0.69 (0.50–0.94)	0.0207*	0.49 (0.27–0.89)	0.0191*	1.15 (0.46–2.98)	0.7562	1.02 (0.53–1.96)	0.9607
TNM stage	I	1		ND		ND		1		1		1		1	
	II	0.58 (0.31–1.08)	0.0837	1		1		0.48 (0.20–1.16)	0.1027	0.25 (0.14–0.45)	<0.0001**	0.97 (0.29–3.23)	0.9641	0.14 (0.03–0.66)	0.0130*
	III	0.20 (0.12–0.35)	<0.0001**	0.39 (0.17–0.92)	0.0315*	0.41 (0.23–0.75)	0.0039**	0.13 (0.02–1.16)	0.0680	0.08 (0.04–0.17)	<0.0001**	0.14 (0.04–0.47)	0.0016**	0.14 (0.06–0.35)	<0.0001**
	IV	ND		0.11 (0.05–0.28)	<0.0001**	0.11 (0.02–0.49)	0.0040**	ND		0.17 (0.02–1.31)	0.0892	ND		0.04 (0.01–0.09)	<0.0001**

Multivariate		Lung		Colon		Stomach		Pancreas		Liver		Breast		Kidney	
		HR	*P* value	HR	*P* value	HR	*P* value	HR	*P* value	HR	*P* value	HR	*P* value	HR	*P* value
Sex	Male	1		1		1		1		1		ND		1	
	Female	1.64 (1.01–2.66)	0.0441*	1.09 (0.52–2.30)	0.3388	0.77 (0.42–1.38)	0.3753	0.88 (0.65–1.20)	0.4215	0.84 (0.43–1.63)	0.6050			1.65 (0.72–3.81)	0.2403
Age (years)	≤65	1		1		1		1		1		1		1	
	>65	0.65 (0.41–1.05)	0.0781	0.42 (0.19–0.93)	0.0321*	0.83 (0.50–1.39)	0.4864	1.03 (0.76–1.41)	0.8370	1.05 (0.63–1.74)	0.8595	0.77 (0.27–2.17)	0.6148	0.65 (0.33–1.28)	0.2099
hTERT	Low	1		1		1		1		1		1		1	
	High	0.61 (0.37–0.97)	0.0376*	0.91 (0.44–1.92)	0.8141	1.49 (0.87–2.56)	0.1461	0.70 (0.51–0.97)	0.0311*	0.48 (0.26–0.87)	0.0148*	0.93 (0.37–2.45)	0.8842	0.82 (0.39–1.73)	0.5984
TNM stage	I	1		ND		ND		1		1		1		1	
	II	0.61 (0.33–1.14)	0.1215	1		1		0.48 (0.20–1.17)	0.1076	0.24 (0.14–0.44)	<0.0001**	0.77 (0.20–3.03)	0.7164	0.12 (0.02–062)	0.0110*
	III	0.20 (0.12–0.35)	<0.0001**	0.32 (0.13–0.76)	0.0101*	0.35 (0.18–0.66)	0.0013**	0.14 (0.02–1.26)	0.0800	0.08 (0.04–0.17)	<0.0001**	0.10 (0.03–0.39)	0.0009**	0.14 (0.06–0.35)	<0.0001**
	IV	ND		0.09 (0.04–0.22)	<0.0001**	0.08 (0.02–0.38)	0.0017**	ND		0.15 (0.02–1.12)	0.0644	ND		0.03 (0.01–0.09)	<0.0001**

HR, hazard ratio; TNM stage, UICC 7th edition; ND, not determined.

* *p <* 0.05;

** *p <* 0.01.

**Table 2:** Univariate and multivariate analyses of disease‐free survival

Univariate		Lung		Colon		Stomach		Pancreas		Liver		Breast		Kidney	
		HR	*P* value	HR	*P* value	HR	*P* value	HR	*P* value	HR	*P* value	HR	*P* value	HR	*P* value
Sex	Male	1		1		1		1		1		ND		1	
	Female	1.20 (0.78–1.86)	0.4084	0.88 (0.43–1.83)	0.7423	1.04 (0.58–1.89)	0.8929	0.82 (0.62–1.09)	0.1678	0.98 (0.68–1.43)	0.9323			0.92 (0.37–2.29)	0.8601
Age	≤65	1		1		1		1		1		1		1	
	>65	1.08 (0.70–1.65)	0.7399	1.13 (0.53–2.34)	0.7455	0.81 (0.48–1.37)	0.4337	1.11 (0.83–1.49)	0.4743	0.80 (0.59–1.08)	0.1429	0.75 (0.39–1.45)	0.3833	1.27 (0.49–3.28)	0.6212
hTERT	Low	1		1		1		1		1		1		1	
	High	0.68 (0.44–1.06)	0.0887	1.40 (0.66–2.95)	0.3825	0.67 (0.39–1.14)	0.1359	0.75 (0.55–1.00)	0.0529	0.67 (0.49–0.92)	0.0137*	0.84 (0.45–1.56)	0.5703	1.14 (0.46–2.83)	0.7756
TNM stage	I	1		ND		ND		1		1		1		1	
	II	0.34 (0.20–0.58)	<0.0001**	1		1		0.33 (0.12–0.89)	0.0287*	0.57 (0.41–0.78)	0.0004**	0.60 (0.27–1.34)	0.2124	0.08 (0.01–0.42)	0.0031*
	III	0.11 (0.07–0.19)	<0.0001**	0.08 (0.02–0.36)	0.0009**	0.32 (0.16–0.64)	0.0013**	0.16 (0.02–1.44)	0.1022	0.24 (0.14–0.40)	<0.0001**	0.17 (0.07–0.43)	0.0002**	0.07 (0.02–0.21)	<0.0001**
	IV	ND		0.02 (0.004–0.08)	<0.0001**	0.17 (0.04–0.78)	0.0228*	0.02 (0.002–0.16)	0.0004**	0.09 (0.06–0.45)	<0.0001**	ND		0.001 (0–0.04)	<0.0001**

Multivariate		Lung		Colon		Stomach		Pancreas		Liver		Breast		Kidney	
		HR	*P* value	HR	*P* value	HR	*P* value	HR	*P* value	HR	*P* value	HR	*P* value	HR	*P* value
Sex	Male	1		1		1		1		1		ND		1	
	Female	1.11 (0.71–1.73)	0.6470	1.39 (0.64–3.05)	0.4051	0.85 (0.46–1.57)	0.5997	0.87 (0.33–0.65)	0.3331	0.94 (0.64–1.38)	0.7561			0.88 (0.35–2.26)	0.7983
Age	≤65	1		1		1		1		1		1		1	
	>65	0.99 (0.94–1.53)	0.9524	0.85 (0.39–1.84)	0.6714	0.75 (0.44–1.29)	0.2968	1.13 (0.84–1.51)	0.4226	0.81 (0.60–1.09)	0.1700	0.80 (0.40–1.63)	0.5463	1.47 (0.53–4.11)	0.4620
hTERT	Low	1		1		1		1		1		1		1	
	High	0.73 (0.47–1.13)	0.1663	1.43 (0.65–3.12)	0.3744	0.82 (0.48–1.42)	0.4815	0.73 (0.54–0.99)	0.0442*	0.67 (0.49–0.92)	0.0135*	0.75 (0.40–1.42)	0.3745	0.94 (0.36–2.44)	0.8915
TNM stage	I	1		ND				1		1		1		1	
	II	0.35 (0.20–0.59)	0.0001**	1		1		0.33 (0.12–0.89)	0.0293*	0.55 (0.40–0.75)	0.0002**	0.50 (0.21–1.19)	0.1182	0.07 (0.01–0.42)	0.0033**
	III	0.12 (0.07–0.20)	<0.0001**	0.08 (0.02–0.34)	0.0006**	0.32 (0.16–0.65)	0.0017*	0.17 (0.02–1.56)	0.1173	0.23 (0.13–0.39)	<0.0001**	0.15 (0.06–0.39)	<0.0001**	0.07 (0.02–0.21)	<0.0001**
	IV	ND		0.02 (0.003–0.07)	<0.0001**	0.17 (0.03–0.84)	0.0297*	ND		ND		ND		ND	

HR, hazard ratio; TNM stage, UICC 7th edition; ND, not determined.

* *p <* 0.05;

** *p* < 0.01.

Overall survival and disease‐free survival were longer for patients whose tumors showed low p‐hTERT expression than for patients whose cancers showed high p‐hTERT expression in cancers of the lung (*p* = 0.0223; *p* = 0.0866), pancreas (*p* = 0.0199; *p* = 0.0494), and liver (*p* = 0.0168; *p* = 0.0128; *p* = 0.0008; *p* = 0.0006; Figure 4B and supplementary material, Figures S5 and S6). These data indicate that high p‐hTERT expression is closely associated with poorer prognosis, independent of TNM stage, supporting its prognostic value in lung, pancreatic, and liver cancers.

### hTERT phosphorylation is associated with aggressive features

Based on our findings that the phosphorylation of hTERT at T249 correlates with poor prognosis in lung, pancreatic, and liver cancers, we further analyzed the clinicopathological characteristics of p‐hTERT in these cancers. Lung and pancreatic cancers with squamous differentiation (adenosquamous carcinoma, an aggressive and immature phenotype [32, 33], and squamous cell carcinoma) demonstrated higher p‐hTERT expression levels than those of adenocarcinoma (*p* = 0.0473; *p <* 0.0001, Figure 4C). Furthermore, the squamous cell makers ΔNp63 and cytokeratin 5/6 (Figure 4D) were associated with p‐hTERT (*p* < 0.01; *p* = 0.02, Figure 4E). A previous report has shown that ΔNp63α induces TERT promoter activation and RNA splicing in mice [34]; therefore, p‐hTERT expression in cancers might be closely associated with squamous differentiation.

Furthermore, p‐hTERT expression was correlated with the invasive type of lung adenocarcinoma (supplementary material, Figure S7A); serum levels of AFP, which is a representative oncofetal protein activated in liver cancer with stem cell properties (supplementary material, Figure S7B); and triple‐negative breast cancer, which is a highly aggressive form of breast cancer with stem cell properties (supplementary material, Figure S7C). In the same cohort, mitosis score (supplementary material, Figure S8) and telomere length were not correlated with overall survival and disease‐free survival (supplementary material, Figure S9). Previously, we have reported that CDK1 phosphorylates hTERT at threonine 249, which represents RdRP activity [16]. hTERT‐RdRP activity involves tumor formation via regulating the expression of target genes. In the present study, we have shown that h‐TERT‐RdRP activity, which is a non‐telomelic function of hTERT, is closely associated with aggressiveness and poorer prognosis via the regulation of proliferation and differentiation in cancer (supplementary material, Figure S10).

## Discussion

Our results clearly indicated that p‐hTERT expression is a strong risk factor independent of TNM stages in lung cancer, pancreatic cancer, and liver cancer, three highly aggressive cancer types with poor prognosis. Moreover, p‐hTERT expression was strongly associated with markers of squamous cell differentiation and aggressive features, suggesting that p‐hTERT expression is a common molecular event in these aggressive cancers. Using a large number of cohorts, we also found that p‐hTERT is correlated with the following parameters: (1) mitotic activity in lung, colon, stomach, pancreatic, and liver cancers; (2) pathological grade (differentiation) in lung, pancreatic, and liver cancers; (3) nuclear score (nuclear pleomorphism) in lung and liver cancers; (4) squamous differentiation in lung and pancreas cancers; and (5) aggressive immature features in lung, pancreatic, liver, and breast cancers, as summarized in supplementary material, Table S3. Our findings suggest the utility of histological evaluations of p‐hTERT expression for prognostic stratification in these deadly cancers.

hTERT canonically regulates telomere lengthening via hTERT recruitment to telomeres at the S‐phase. However, we have previously demonstrated that it exhibits RdRP activity at the M‐phase [14], and this non‐canonical hTERT function is acquired by CDK1‐mediated phosphorylation [16]. In this study, we identified a positive correlation between p‐hTERT expression and the incidence of mitosis in our cancer panels, indicating that hTERT‐RdRP is activated in various human cancers with high frequencies of mitotic cells. Furthermore, p‐hTERT expression did not show a clear association with telomere length, suggesting that immunoreactivity of the newly developed monoclonal antibody reflects RdRP activity rather than telomerase activity.

We found that p‐hTERT expression has prognostic value in lung, pancreatic, and liver cancers but not in colon and stomach cancers. Interestingly, although p‐hTERT expression increased as the mitosis score increased in all of these cancers, p‐hTERT expression decreased as the pathological grade increased in colon and stomach cancers. It is unclear why the relationships between p‐hTERT expression, mitosis scores, and pathological scores differed according to the cancer origin. We found that cancer cells in the mucosal layer showed higher expression levels of p‐hTERT than those in the muscular or subserosal layer in colon and stomach cancers (unpublished data). It is possible that additional mechanisms regulate p‐hTERT expression, potentially related to the tumor microenvironment, in the mucosal layer of cancer originating in luminal organs.

Although p‐hTERT expression is associated with immature and aggressive pathological/clinical features in several cancers, correlation with poor prognostic outcome was only shown in lung, pancreatic, and liver cancers. One possibility to explain the different clinical impacts of p‐hTERT expression on prognosis in distinct cancer subtypes would be the surgical procedures and curability. Curative surgical resection is generally achieved in limited cases of lung, liver, and pancreatic cancers, potentially due to the microscopic tumor dissemination at the time of surgery, resulting in early recurrence with poor prognosis. In contrast, radical resection could be technically achieved irrespective of immature and aggressive features in some colon and stomach cancers. In these cancers, the value of p‐hTERT expression evaluation might be limited. Another possibility is that more impactful genetic alteration or molecular abnormality, in addition to p‐hTERT phosphorylation, might exist and determine the patients' prognosis in colon, stomach, and breast cancers. To clarify the prognostic utility of p‐hTERT in low‐risk patients, therefore, we have analyzed p‐hTERT expression in pancreatic neuroendocrine tumor and found an association between p‐hTERT expression and large tumor size and high mitotic activity, suggesting that p‐hTERT might be a marker of cancers with aggressive features even in low‐risk localized disease (unpublished data).

We have also analyzed the intensity and H‐score of phosphorylation of hTERT 249T of most of the cases (83 hepatocellular carcinoma cases were not analyzed). However, only high H‐score was correlated with poor prognosis in lung cancer but not in other cancers. The percentage of phosphorylation of hTERT showed the most significant correlation with prognosis in lung, pancreas, and liver cancers as compared with the intensity and H‐score. Evaluation of the percentage of p‐hTERT might be the best approach in a clinical setting.

Our results indicate that the evaluation of p‐hTERT could be utilized for prognostic stratification in clinical settings. Furthermore, our monoclonal antibody can detect p‐hTERT in formalin‐fixed, paraffin‐embedded samples using an automatic immunostaining system, thus providing a basis for the development of a novel clinical diagnostic tool to identify patients with aggressive cancer.

## Author contributions statement

YMa, JY and NM curated the data. YMa, YK and KM acquired funding. JY, MY, KY, YMu, MM, SY, JT and MKa carried out the investigation. YMa, TYa, YK and KM were responsible for methodology, and YD, YMi, TYo, TO, HI, SM, TK, TM, MKo, SK, TM, MH, RH, KK, NK and KO for resources. YMa was responsible for software. YMa, TYa and KM supervised the study. YMa wrote the original draft, and YMa, TYa and KM reviewed and edited the final manuscript.

## Supporting information


**Supplementary materials and methods** 
**Figure S1.** p‐hTERT expression is not associated with TNM stage
**Figure S2.** p‐hTERT expression is not associated with age
**Figure S3.** p‐hTERT expression is not associated with sex
**Figure S4.** Positivity of Ki67 was associated with mitosis
**Figure S5.** p‐hTERT and survival
**Figure S6.** p‐hTERT and survival of liver cancer cohorts
**Figure S7.** (A) Noguchi classification and p‐hTERT in lung cancer. (B) Serum AFP levels and p‐hTERT in liver cancer. (C) Triple‐negative breast cancer and p‐hTERT
**Figure S8.** Mitosis score and survival
**Figure S9.** Telomere length and survival
**Figure S10.** Non‐telomeric and telomeric function of hTERT
**Table S1.** Human cell lines used
**Table S2.** Clinicopathological characteristics of patients with high and low hTERT phosphorylation
**Table S3.** Clinicopathological factors related to high levels of p‐hTERT
