# Supplementary material for: Phosphorylation of hTERT at threonine 249 is a novel tumor biomarker of aggressive cancer with poor prognosis in multiple organs
Source: J Pathol. 2022 Mar 23;257(2):172–85. doi: 10.1002/path.5876 (PMC9315154; doi:10.1002/path.5876)
Supplement: Supplementary file 1 — Supplementary materials and methods Figure S1. p‐hTERT expression is not associated with TNM stage Figure S2. p‐hTERT expression is not associated with age Figure S3. p‐hTERT expression is not associated with sex Figure S4. Positivity of Ki67 was associated with mitosis Figure S5. p‐hTERT and survival Figure S6. p‐hTERT and survival of liver cancer cohorts Figure S7. (A) Noguchi classification and p‐hTERT in lung cancer. (B) Serum AFP levels and p‐hTERT in liver cancer. (C) Triple‐negative breast cancer and p‐hTERT Figure S8. Mitosis score and survival Figure S9. Telomere length and survival Figure S10. Non‐telomeric and telomeric function of hTERT Table S1. Human cell lines used Table S2. Clinicopathological characteristics of patients with high and low hTERT phosphorylation Table S3. Clinicopathological factors related to high levels of p‐hTERT [file PATH-257-172-s001.docx]

**Phosphorylation of hTERT at threonine 249 is a novel tumor biomarker of aggressive cancer with poor prognosis in multiple organs**

Y Matsuda *et al. J Pathol* DOI: 10.1002/path.5876

**Supplementary materials and methods**

Reference numbers refer to the main text list

***Hybridoma production***

Female 4-week-old BALB/c mice were purchased from CLEA Japan (Tokyo, Japan) and kept under specific pathogen-free conditions. The Animal Care and Use Committee of Tohoku University approved all animal experiments described in this study. BALB/c mice were immunized by intraperitoneal (i.p.) injection of 100 μg of the phosphorylated hTERT (p-hTERT) peptide together with Imject Alum (Thermo Fisher Scientific Inc, Waltham, MA, USA). After two additional immunizations of 100 μg, an i.p. booster injection of 100 μg was given 2 days before spleen cells were harvested. The spleen cells were fused with P3U1 cells (ATCC, Manassas, VA, USA) using PEG1500 (Roche Diagnostics, Indianapolis, IN, USA). The hybridomas were grown in RPMI 1640 medium (Nacalai Tesque, Inc, Kyoto, Japan) at 37 °C in a humidified atmosphere containing 5% CO_2_ and 95% air, supplemented with 10% heat-inactivated fetal bovine serum (Thermo Fisher Scientific Inc), hypoxanthine, aminopterin, and thymidine selection medium supplement (Thermo Fisher Scientific Inc), and 5% BriClone Hybridoma Cloning Medium (QED Bioscience Inc, San Diego, CA, USA). Then 100 units/ml penicillin, 100 μg/ml streptomycin, and 0.25 μg/ml amphotericin B (Nacalai Tesque, Inc) were added to the culture medium. Plasmocin (5 μg/ml; InvivoGen, San Diego, CA, USA) was added to prevent *Mycoplasma* contamination. Culture supernatants were screened by enzyme-linked immunosorbent assays (ELISAs) for the detection of the p-hTERT peptide and wild-type TERT peptide. Clone TpMab-3 (IgG_1_, kappa), which is specific for the p-hTERT peptide, was finally established.

##### ***ELISA***

Peptides were immobilized on Nunc MaxiSorp 96-Well Immunoplates (Thermo Fisher Scientific Inc) at 1 μg/ml. After blocking with SuperBlock T20 phosphate-buffered saline blocking buffer (Thermo Fisher Scientific Inc), the plates were incubated with culture supernatant with 1:2000 diluted peroxidase-conjugated anti-mouse immunoglobulins (Agilent Technologies Inc, Santa Clara, CA, USA). The enzymatic reaction was conducted using 1-Step Ultra TMB-ELISA (Thermo Fisher Scientific Inc). The optical density was measured at 655 nm using an iMark microplate reader (Bio-Rad Laboratories, Inc, Berkeley, CA, USA). Reactions were performed with a volume of 50–100 μl at 37 °C.

***Detection of phosphorylated hTERT by immunoprecipitation***

Anti-hTERT mouse monoclonal antibodies (mAbs, clones 10E9-2 and 2E4-2) were generated, and the specificity was evaluated as reported previously [15,21]. Approximately 1 × 10^7^ cells were lysed in 1 ml of Lysis Buffer A [0.5% NP-40, 20 mm Tris–HCl (pH 7.4), and 150 mm NaCl]. After sonication, lysates were cleared of insoluble material by centrifugation at 21 000 × *g* at 4 °C for 15 min. One milliliter of lysate was supplemented with 40 μl of Pierce Protein A Plus Agarose (Thermo Fisher Scientific Inc) for 30 min at 4 °C. The pre-absorbed lysate was mixed with 10 μg of anti-hTERT mAb (clone 10E9-2) and 40 μl of Pierce Protein A Plus Agarose, and incubated overnight at 4 °C. Immune complexes were washed three times with Lysis Buffer A, eluted in 2× SDS loading buffer [2% β-mercaptoethanol, 20% glycerol, 4% SDS, and 100 mm Tris–HCl (pH 6.8)], and separated by SDS-PAGE on 8% polyacrylamide gels.

Anti-hTERT mouse mAb (clone 2E4-2, 10 µg/ml) and Mouse TrueBlot ULTRA Anti-Mouse Ig HRP (1:4000; Rockland, Gilbertsville, PA, USA) were used for immunoblotting to detect whole-hTERT proteins. Anti-phospho-hTERT mouse monoclonal Ab (clone TpMab-3, 10 µg/ml) and Mouse TrueBlot ULTRA Anti-Mouse Ig HRP (1:4000; Rockland) were used to detect phosphorylated hTERT. For λ-phosphatase treatment, the bead suspension with immune complexes was treated with 2000 U of λ-protein phosphatase (Bio Academia, Osaka, Japan) and 2 mm MnCl_2_ in λ-PPase reaction buffer [50 mm Tris–HCl (pH 7.6), 100 mm NaCl, 2 mm DTT, 100 μm EDTA, and 0.01% Brij 35] and incubated at 30 °C for 30 min.

***Immunoprecipitation–RdRP assay***

hTERT was immunoprecipitated from human cell lines as described previously with an anti-hTERT mAb (clone 10E9-2) [15,21]. The bead suspension with immune complexes was washed four times with 1× acetate buffer [10 mm HEPES–KOH (pH 7.8), 100 mm potassium acetate, and 4 mm MgCl_2_] containing 10% glycerol, 0.1% Triton-X, and 0.06× cOmplete EDTA-free (Roche Diagnostics, Indianapolis, IN, USA), and once with AGC solution (1× acetate buffer containing 10% glycerol and 0.02% CHAPS) containing 2 mm CaCl_2_. The bead suspension was treated with 0.25 units/μl Micrococcal Nuclease (Takara Bio, Shiga, Japan) at 25 °C for 15 min. Immunoprecipitates were subsequently washed twice with AGC solution containing 3 mm EGTA and once with 1× acetate buffer containing 0.02% CHAPS. Forty microliters of the reaction mixture was prepared by combining 20 μl of the bead suspension with 6 μl of [α-32P] UTP (3000 Ci/mmol) and 25 ng/μl (final concentration) RNA template, followed by incubation at 32 °C for 2 h. The sequence of the RNA template was as follows: 5'-GGGAUCAUGUGGGUCCUAUUACAUUUUAAACCCA-3'. This RNA has hydroxyl groups at both the 5' and the 3' ends. The final concentrations of ribonucleotides were 1 mm ATP, 0.2 mm GTP, 10.5 μm UTP, and 0.2 mm CTP. The products were treated with Proteinase K to stop the reaction, purified several times with phenol/chloroform until the white interface disappeared, and precipitated using ethanol. The RdRP products were treated with RNase I (2 U; Promega, Madison, WI, USA) at 37 °C for 2 h to digest single-stranded RNAs completely, followed by Proteinase K treatment, phenol/chloroform purification, and ethanol precipitation. The products were electrophoresed in a 15% polyacrylamide gel containing 7 m urea and detected by autoradiography.

***Immunofluorescence cell staining***

Cells were fixed with 4% formaldehyde in PBS, permeabilized with 0.2% Triton X-100 in PBS, and blocked with 2% bovine serum albumin in PBS. The cells were incubated with 10 μg/ml of the anti-phospho-hTERT mouse mAb (clone TpMab-3), followed by incubation in the presence of Alexa488-labeled secondary antibody (1:1000; Thermo Fisher Scientific Inc). The nuclei were stained with Hoechst 33342 (NucBlue; Thermo Fisher Scientific Inc).


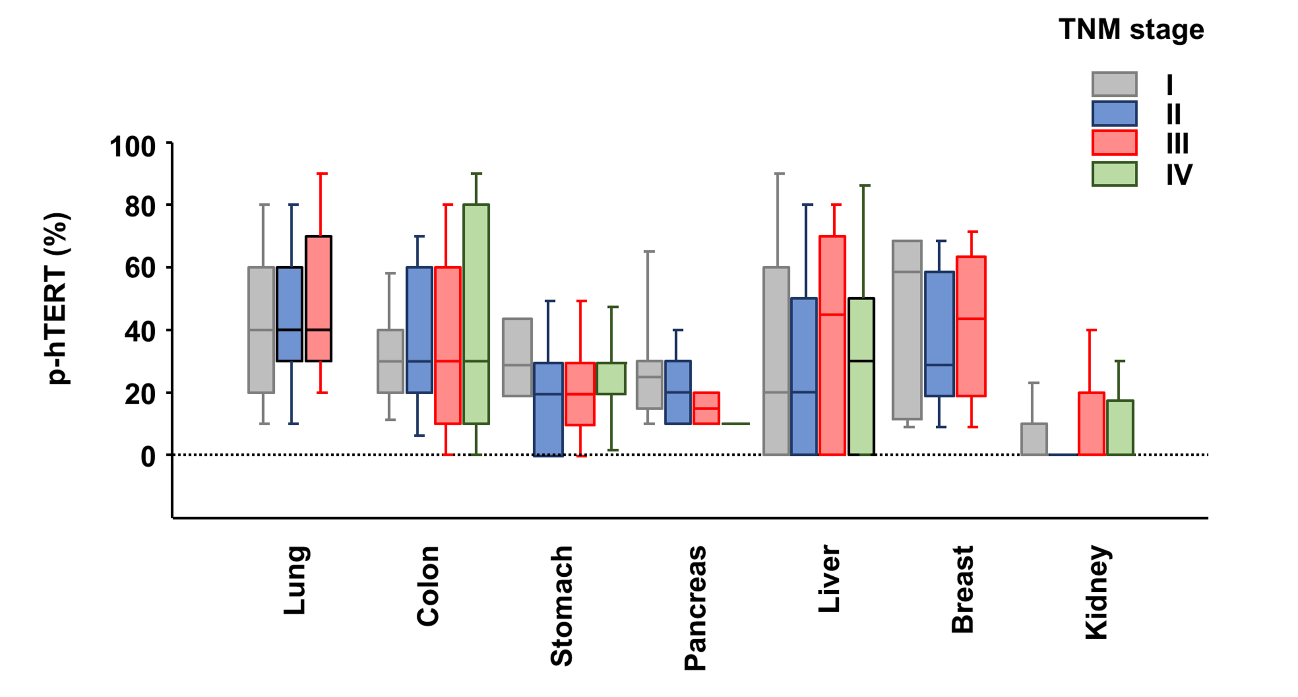


**Figure S1.** p-hTERT expression is not associated with TNM stage. Spearman’s rank correlation coefficient.


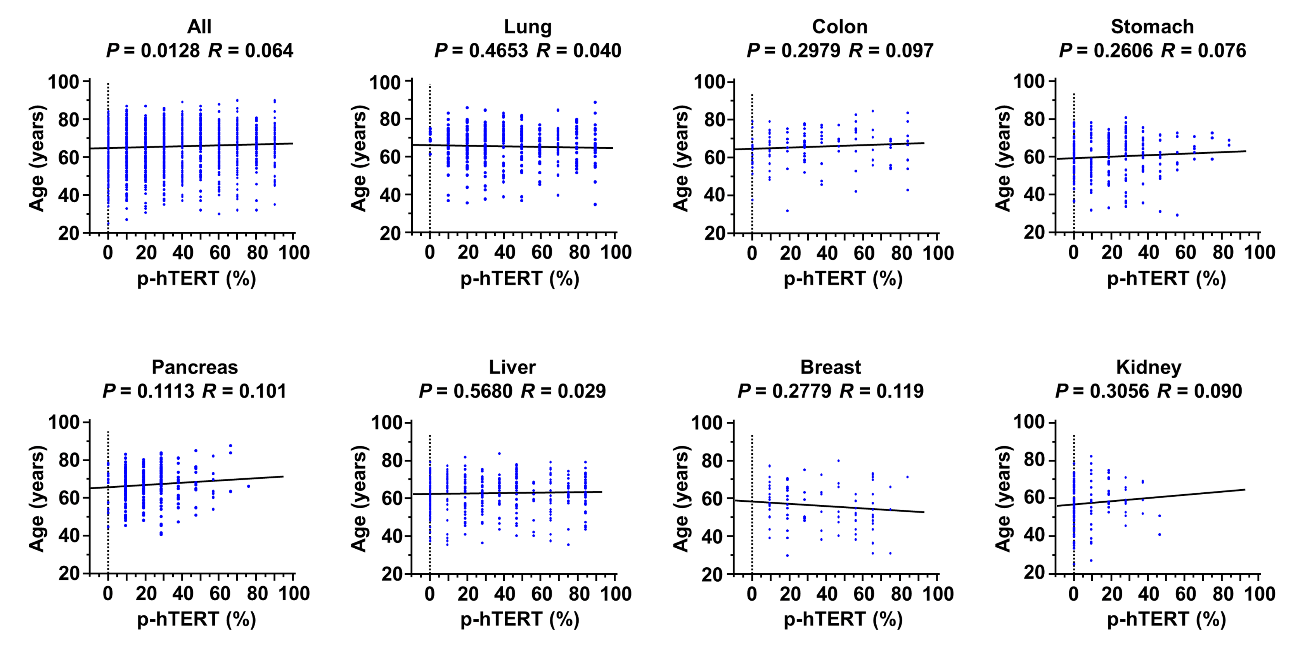


**Figure S2.** p-hTERT expression is not associated with age. Correlation coefficient analysis.


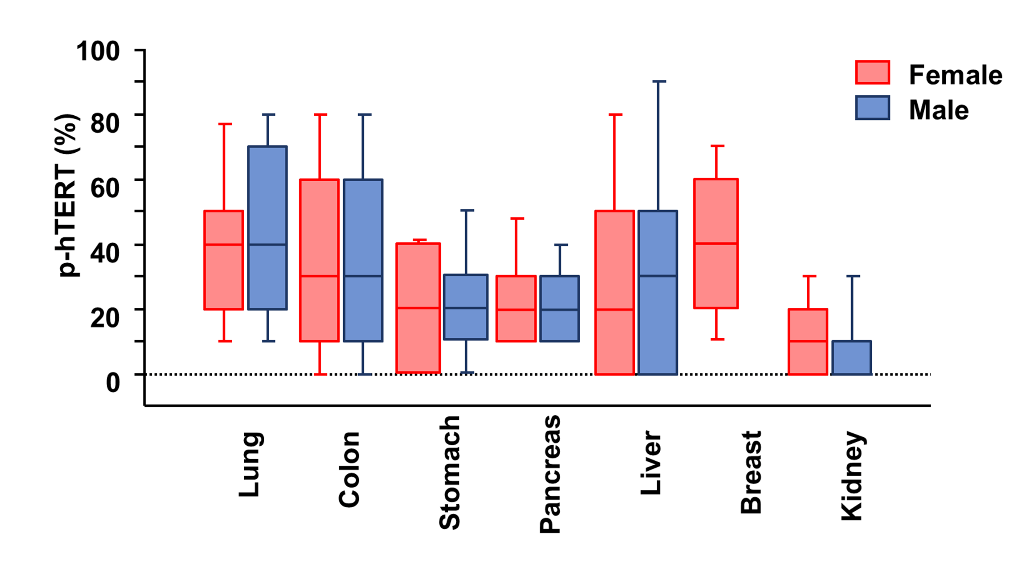


**Figure S3.** p-hTERT expression is not associated with sex. Student’s *t*-test.


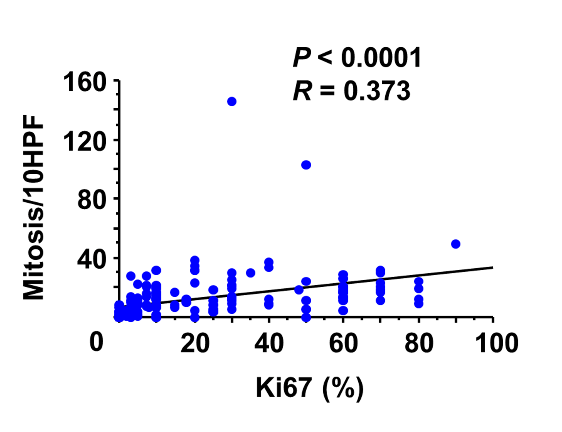


**Figure S4.** Positivity of Ki67 was associated with mitosis. Correlation coefficient analysis.

**Figure S5.** p-hTERT and survival. (A) Overall survival. (B) Disease-free survival. The number of patients at risk is shown below the graph. **p* < 0.05 and ***p* < 0.01 by log-rank test.

**
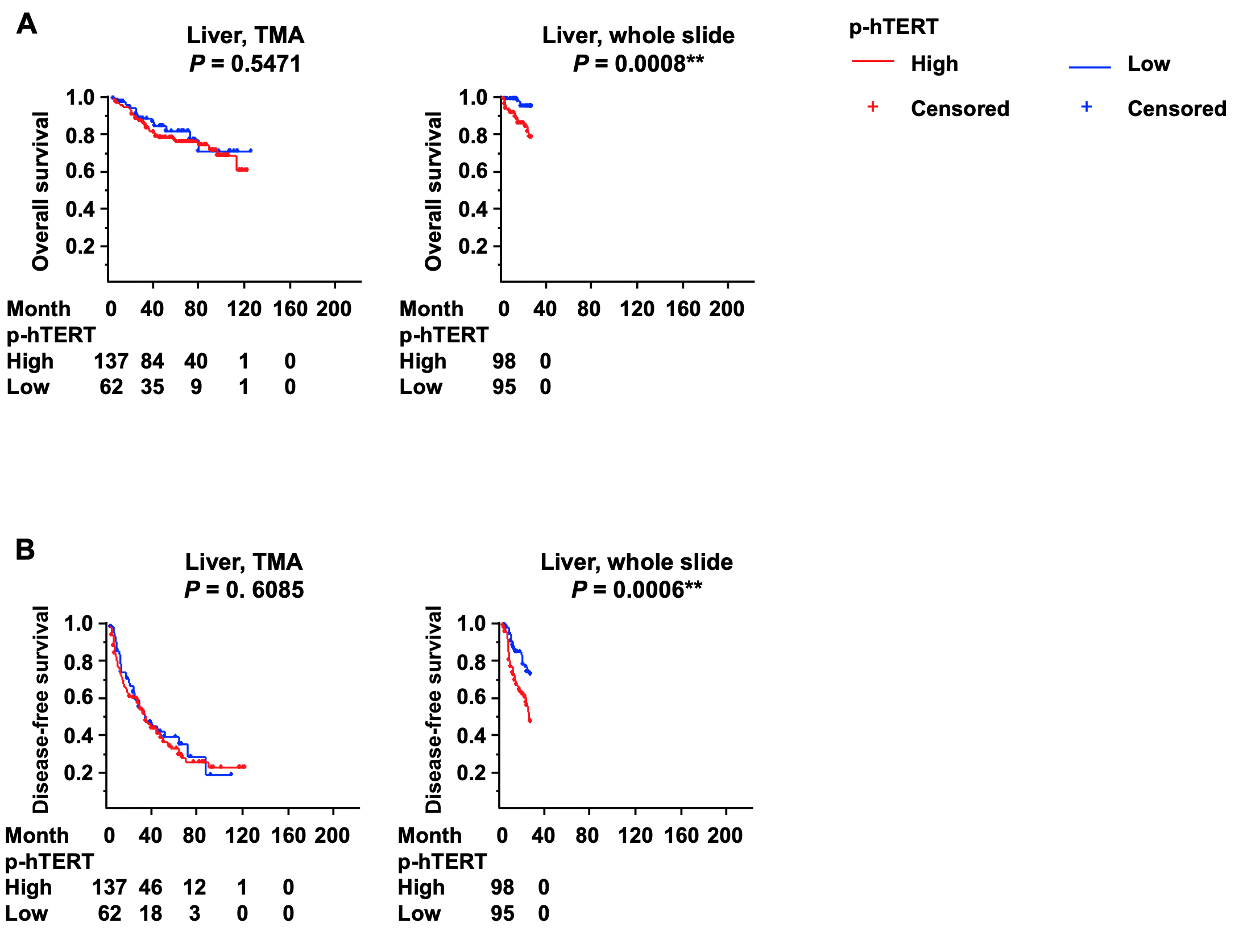
Figure S6.** p-hTERT and survival of liver cancer cohorts. (A) Overall survival. (B) Disease-free survival. The number of patients at risk is shown below the graph. **p* < 0.05 and ***p* < 0.01 by log-rank test.


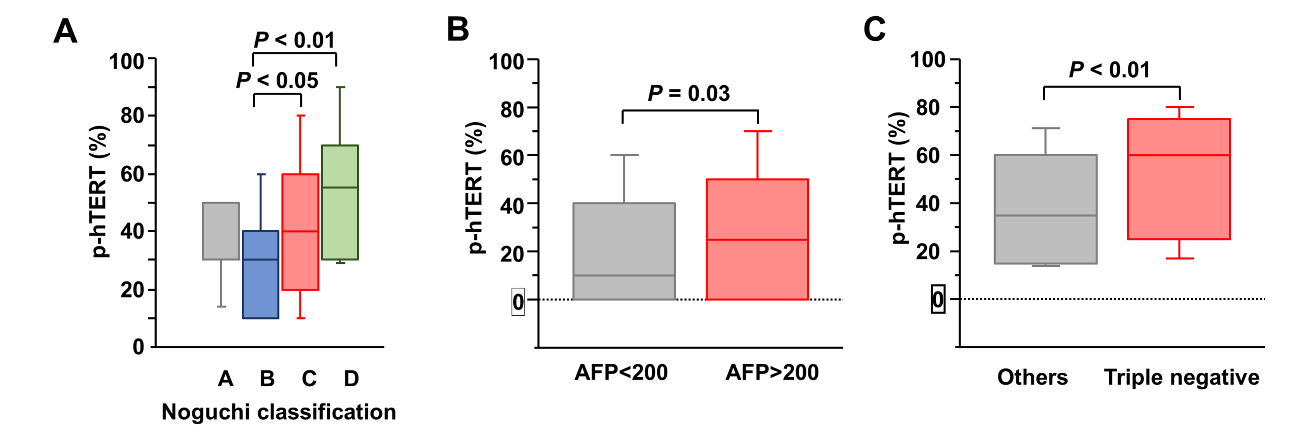


**Figure S7.** (A) Noguchi classification and p-hTERT in lung cancer. (B) Serum AFP levels and p-hTERT in liver cancer. (C) Triple-negative breast cancer and p-hTERT.

**Figure S8.** Mitosis score and survival. (A) Overall survival. (B) Disease-free survival. The number of patients at risk is shown below the graph. ***p* < 0.01 by log-rank test.

**Figure S9.** Telomere length and survival. (A) Overall survival. (B) Disease-free survival. Long and short telomeres were defined as follows: [normalized telomere length of cancer cells]/[normalized telomere length of fibroblasts] with a cut-off value of 1.2 to obtain cases with short telomeres (under 1.2) and cases with long telomere lengths (over 1.2). The number of patients at risk is shown below the graph. Log-rank test.


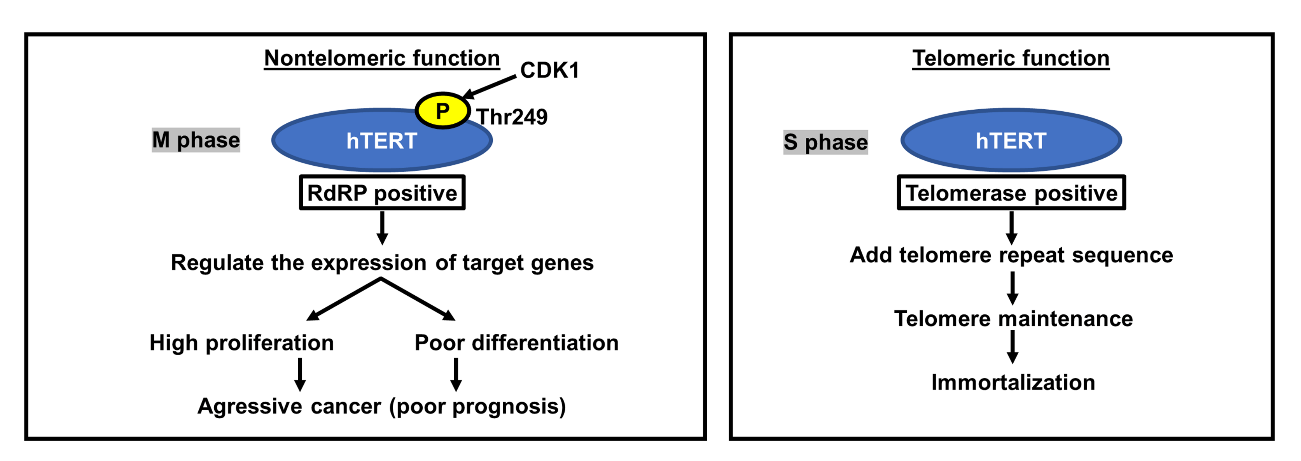


**Figure S10.** Non-telomeric and telomeric function of hTERT.

**Table S1.** Human cell lines used

| **Cell** | **Cell type** | **Origin** | **Medium** | **Source** |
| --- | --- | --- | --- | --- |
| BJ | Normal diploid fibroblast | Skin | KnockOut DMEM/Medium 199 (4:1) with 15% FBS and L-glutamine | ATCC (Manassas, VA, USA) |
| HeLa | Epithelial cancer | Uterine cervix | DMEM with 10% FBS |  |
| 1301 | T-cell leukemia | Blood | RPMI 1640 with 10% FBS | Merck KGaA (Darmstadt, Germany) |
| HFL-1 | Normal diploid fibroblast | Lung | RPMI 1640 with 10% FBS | Japanese Cancer Research Resources Bank (Osaka, Japan) |
| KYSE850 | Squamous cell carcinoma | Esophagus | RPMI 1640/Ham’s F12 (1:1) with 2% FBS |  |
| HLE | Hepatocellular carcinoma | Liver | DMEM with 10% FBS |  |
| HEPG2 | Hepatocellular carcinoma | Liver | MEM with 10% FBS and 1% NEAA |  |
| SSP25 | Cholangiocarcinoma | Liver | RPMI 1640 with 10% FBS |  |
| PANC-1 | Adenocarcinoma | Pancreas | RPMI 1640 with 10% FBS | Cell Resource Center for Biomedical Research, Institute of Development, Aging and Cancer, Tohoku University (Miyagi, Japan) |
| MIAPaCa-2 | Adenocarcinoma | Pancreas | RPMI 1640 with 10% FBS |  |

**Table S2.** Clinicopathological characteristics of patients with high and low hTERT phosphorylation

|  | p-hTERT | Lung | Colon | Stomach | Pancreas | Liver | Breast | Kidney |
| --- | --- | --- | --- | --- | --- | --- | --- | --- |
| Number of patients, *n* (%) |  | 342 (100) | 117 (100) | 202 (100) | 252 (100) | 393 (100) | 85 (100) | 132 (100) |
| TMA, *n* (%) |  | 342 | 117 | 122 | 199 | 199 | 0 | 132 |
| Whole slide, *n* (%) |  | 0 | 0 | 80 | 53 | 194 | 85 | 0 |
| Cut-off value for hTERT |  | 40 | 30 | 20 | 20 | 20 | 40 | 10 |
| Number of patients, *n* (%) | High | 183 (53.5) | 74 (63.2) | 118 (58.4) | 158 (62.7) | 236 (60.1) | 44 (51.8) | 50 (37.9) |
|  | Low | 159 (46.5) | 43 (36.8) | 84 (41.6) | 94 (37.3) | 157 (39.9) | 41 (48.2) | 82 (62.1) |
| Age, years, median (range) | High | 68 (35–90) | 72.5 (44–90) | 66 (30–86) | 69 (40–90) | 69 (37–87) | 57 (32–85) | 59 (25–87) |
|  | Low | 68 (36–87) | 69 (33–84) | 62 (33–83) | 68 (44–85) | 65 (37–81) | 60 (31–82) | 62 (36–84) |
| Sex, male/female, *n* (%) | High | 106 (31.0)/77 (22.5) | 40 (34.2)/34 (29.1) | 86 (42.6)/32 (15.8) | 84 (33.3)/74 (29.4) | 190 (48.3)/46 (11.7) | 0 (0)/44 (51.8) | 30 (22.7)/20 (15.2)* |
|  | Low | 88 (25.7)/71 (20.8) | 23 (19.7.7)/20 (17.1) | 62 (30.7)/22 (10.9) | 61 (24.2)/33 (13.1) | 125 (31.8)/32 (8.1) | 0 (0)/41 (48.2) | 63 (47.7)/18 (13.6) |
| Pathological type, *n* (%) | High | ADC 139 (40.6)/SCC 35 (10.2)/ASC 9 (2.6)/other 0 (0)** | ADC 74 (63.2) | ADC 118 (58.4) | ADC 140 (55.6)/ASC 18 (7.1) | HCC 236 (60.1) | ADC [IDC-NOS 40 (47.1)/other 4 (4.7)] | RCC [Clear 40 (30.3)/Chromo 4 (3.0)/Pap 3 (2.3)/other 3 (2.3)] |
|  | Low | ADC 143 (41.8)/SCC 11 (3.2)/ASC 2 (0.6)/other 3 (0.9) | ADC 43 (36.8) | ADC 84 (41.6) | ADC 89 (35.3)/ASC 5 (2.0) | HCC 157 (39.9) | ADC [IDC-NOS 36 (42.4)/other 5 (5.9)] | RCC [Clear 69 (52.3)/Chromo 2 (1.5)/Pap 4 (3.0)/other 7 (5.3)] |
| Mitosis score, 1/2/3, *n* (%) | High | 139 (40.6)/25 (7.3)/19 (5.6)** | 26 (22.2)/26 (22.2)/22 (18.8)* | 59 (29.2)/26 (12.9)/33 (16.3)** | 131 (52.0)/19 (7.5)/8 (3.2)* | 194 (49.4)/25 (6.4)/17 (4.3)** | 28 (33.0)/12 (14.1)/4 (4.7) | 50 (37.9)/0 (0.0)/0 (0.0) |
|  | Low | 142 (41.5)/8 (2.3)/9 (2.6) | 26 (22.2)/10 (8.5)/7 (6.7) | 61 (30.2)/12 (5.9)/11 (5.4) | 89 (35.3)/4 (1.6)/1 (0.4) | 147 (37.4)/6 (1.5)/4 (1.0) | 28 (32.9)/8 (9.4)/5 (5.9) | 81 (61.4)/0 (0.0)/1 (0.8) |
| Pathological grade, G1/G2/G3/G4, *n* (%) | High | 23 (6.8)/100 (29.2)/51 (14.9)/9 (2.6)** | 30 (25.6)/40 (34.2)/4 (3.4)/0 (0) | 25 (12.4)/32 (15.8)/61 (30.2)/0 (0) | 54 (21.4)/69 (27.4)/17 (6.7)/18 (7.1)** | 23 (5.9)/183 (46.6)/30 (7.6)/0 (0)** | 18 (21.2)/23 (27.1)/3 (3.5) | 10 (7.6)/16 (12.1)/15 (11.4)/9 (6.8) |
|  | Low | 39 (11.4)/90 (26.3)/25 (7.3)/5 (1.5) | 11 (9.4)/25 (21.4)/7 (6.0)/0 (0) | 8 (4.0)/26 (12.9)/50 (24.8)/0 (0) | 53 (21.0)/33 (13.1)/4 (1.6)/4 (1.6) | 27 (6.9)/123 (31.3)/7 (1.8)/0 (0) | 23 (27.1)/14 (16.5)/4 (4.7) | 9 (6.8)/39 (29.5)/28 (21.2)/6 (4.5) |
| Nuclear score, 1/2/3, *n* (%) | High | 36 (10.5)/143 (41.8)/4 (1.2)** | 3 (2.6)/71 (60.7)/0 (0) | 7 (3.5)/107 (53.0)/4 (2.0) | 0 (0.0)/145 (57.5)/13 (5.2) | 83 (21.1)/138 (35.1)/15 (3.8) | 5 (5.9)/28 (32.9)/11 (12.9) | 21 (15.9)/27 (20.5)/2 (1.5) |
|  | Low | 55 (16.1)/101 (29.5)/3 (0.9) | 1 (0.9)/41 (35.0)/1 (0.9) | 10 (5.0)/73 (36.1)/1 (0.5) | 1 (0.4)/88 (34.9)/5 (2.0) | 71 (18.1)/80 (20.4)/6 (1.5) | 7 (8.2)/22 (25.9)/11 (12.9) | 38 (28.8)/40 (30.3)/4 (3.0) |
| TNM stage, I/II/III/IV, *n* (%) | High | 135 (39.5)/27 (7.9)/21 (6.1)/0 (0.0) | 4 (3.4)/33 (28.2)/25 (21.4)/10 (8.5), ND=2 | 3 (1.5)/36 (17.8)/65 (32.2)/14 (6.9) | 6 (2.4)/151 (59.9)/1 (0.4)/0 (0.0) | 136 (34.6)/78 (19.8)/18 (4.6)/4 (1.0) | 14 (16.5)/22 (25.9)/8 (9.4)/0 (0.0) | 32 (23.5)/0 (0.0)/11 (8.3)/7 (5.3) |
|  | Low | 125 (36.5)/23 (6.7)/11 (3.2)/0 (0.0) | 2 (1.7)/18 (15.4)/17 (14.5)/6 (5.1) | 1 (0.5)/34 (16.8)/46 (22.8)/3 (1.5) | 2 (0.8)/90 (35.7)/1 (0.4)/1 (0.4) | 93 (23.7)/54(13.7)/8 (2.0)/2 (0.5) | 9 (10.6)/28 (32.9)/4 (4.7)/0 (0.0) | 50 (37.9)/4 (3.0)/16 (12.2)/12 (9.1) |
| Relative telomere length, cut-off 1.2, long/short, *n* (%) | High | 17 (5.0)/164 (48.0), ND=2 | 0 (0)/74 (63.2) | 12 (5.9)/101 (50.0), ND=5 | 25 (9.9)/130 (51.6), ND=3 | 5 (1.3)/198 (50.4), ND=33 | 5 (5.9)/35 (41.2), ND=4 | 9 (6.8)/41 (31.1) |
|  | Low | 7 (2.0)/149 (43.6), ND=3 | 0 (0)/43 (36.7) | 10 (5.0)/68 (33.7), ND=6 | 9 (3.6)/74 (29.4), ND=11 | 0 (0)/105 (26.7), ND=52 | 7 (8.2)/30 (35.3), ND=4 | 10 (7.6)/71 (53.8), ND=1 |

TMA, tissue microarray; ADC, adenocarcinoma; SCC, squamous cell carcinoma; ASC, adenosquamous carcinoma; HCC, hepatocellular carcinoma; IDC-NOS, intraductal carcinoma, not specific; Apo, apocrine carcinoma; RCC, renal cell carcinoma; clear, clear cell renal cell carcinoma; chromo, chromophobe renal cell carcinoma; pap, papillary renal cell carcinoma; TNM stage, UICC 7th edition; ND, not determined. **p* < 0.05; ***p* < 0.01.

**Table S3.** Clinicopathological factors related to high levels of p-hTERT

| **Organ** |  |  |  |  |  |
| --- | --- | --- | --- | --- | --- |
| Lung | High mitosis | High pathological grade | Strong nuclear atypia | Invasive feature | Short OS |
| Colon | High mitosis |  |  |  |  |
| Stomach | High mitosis |  |  |  |  |
| Pancreas | High mitosis | High pathological grade | Short OS |  |  |
| Liver | High mitosis | High AFP | Short OS |  |  |
| Breast | Triple negative | |  |  |  |
| Kidney |  |  |  |  |  |
